# Radiologic Outcomes after Operative Management of Traumatic Spine Fractures: Stand-Alone Posterior Stabilization versus Combined Anteroposterior Approach

**DOI:** 10.1055/a-2331-2466

**Published:** 2024-06-21

**Authors:** Ali Mulhem, Ziad Omran, Stefanie Hammersen, Sven Rainer Kantelhardt

**Affiliations:** 1 Department for Continuing Education, University of Oxford, Oxford, United Kingdom of Great Britain and Northern Ireland; 2 Department of Neurosurgery, Vivantes Klinikum im Friedrichshain, Berlin, Germany

**Keywords:** spine fracture, sagittal alignment, vertebral height, comparative study, surgical approach

## Abstract

**Background:**

Previous research emphasizes correcting deformities resulting from spine fractures by restoring sagittal alignment and vertebral height. This study aims to compare radiologic outcomes, including sagittal index (SI) and loss of vertebral body height (LVBH), between stand-alone posterior stabilization (group I) and the posteroanterior/combined approach (group II) in the operative management of traumatic thoracic or lumbar spine fractures.

**Methods:**

In this retrospective single-center study, all patients with traumatic spine fractures (T1–L5) undergoing surgical stabilization between January 1, 2015, and May 31, 2021, were included. Two spine surgeons independently assessed imaging, recording the SI and LVBH values at baseline, after each surgical intervention, and during follow-up (at least 3 months posttreatment). The mean SI and LVBH values between the assessing surgeons were utilized. Linear mixed-effects regression models, adjusted to baseline values, compared the SI and the LVBH values between the two groups.

**Results:**

In all, 71 patients (42 men), with the median age of 38 years (interquartile range [IQR]: 28–54) and median follow-up of 4 months (IQR: 3–17), were included. Thirty-two patients were in group I and 39 patients were in group II. Forty fractures included the thoracolumbar junction (T12 or L1), 15 affected the thoracic spine, and 14 the lumbar spine. The regression model revealed superior sagittal alignment in group II, with an adjusted mean difference for SI of –4.24 (95% confidence interval [CI]: –7.13 to –1.36; *p* = 0.004), and enhanced restoration of vertebral body height with an adjusted mean difference for LVBH of 0.11 in the combined approach (95% CI: 0.02–0.20; *p* = 0.02). Nine postoperative complications occurred in the entire cohort (4 in group I and 5 in group II).

**Conclusions:**

Combined posteroanterior stabilization for spine fractures improves deformities by enhancing sagittal alignment and increasing vertebral body height, with acceptable morbidity compared with the stand-alone posterior approach.

## Introduction

Severe spinal fractures impose substantial medical, social, and economic burdens, particularly given their predilection for adolescents and young adults, affecting their crucial productive years. 1 These fractures predominantly manifest in the thoracic and thoracolumbar regions, often stemming from motor vehicle accidents, falls from heights, and, notably, instances of suicidal jumping. 2

The management of such fractures typically involves two primary surgical approaches: the stand-alone dorsal approach employing transpedicular screw rod fixation with or without implant removal after consolidation of fractures and the posteroanterior combined approach, wherein dorsal stabilization is reinforced by anterior fixation through vertebral body replacement. 3 Controversy surrounds the determination of the optimal surgical treatment. 3

To comprehensively assess surgical outcomes, numerous authors advocate evaluating short-term clinical results and scrutinizing the correction of deformities arising from the fractures, specifically sagittal alignment and reduction in vertebral height. Previous studies have underscored a positive correlation between sagittal index (SI) and loss of vertebral height (LVH) with long-term clinical outcomes. 3 4 5 Patients with an increased SI (indicative of malalignment) and heightened LVH (suggesting increased compression) are more prone to experiencing or developing severe neurologic injuries compared with those with less deformity. 3 6

Therefore, this study aims to compare radiologic outcomes and complications following surgical treatment of traumatic spine fractures between stand-alone posterior stabilization versus anteroposterior stabilization (combined approach).

## Methods and Materials

### Patients Selection

We conducted a retrospective review of patient records and imaging studies for all patients treated at our supra-regional maximal care trauma center for traumatic spine fractures between January 1, 2015, and May 31, 2021. Patients were identified using International Classification of Diseases, 10th revision (ICD-10) codes S22.0 and S32.0.

Patients were included based on the following eligibility criteria:

- Patients with a spine fracture at any vertebrae from the first thoracic (T1) to the last lumbar (L5).
- Fractures diagnosed after a specified date of trauma, including traffic accidents, falls from a height (suicidal or non-suicidal), or other acute traumatic insults.
- No age restriction.
- Only patients treated surgically by either a posterior or combined approach were included. The surgical approach was chosen based on the preference of the attending neurosurgeon.
- Patients with cervical fractures, pathologic fractures (due to tumor or metastasis) or patients with a previous history of osteoporosis were excluded.

### Patients Groups

**Fig. 1:**
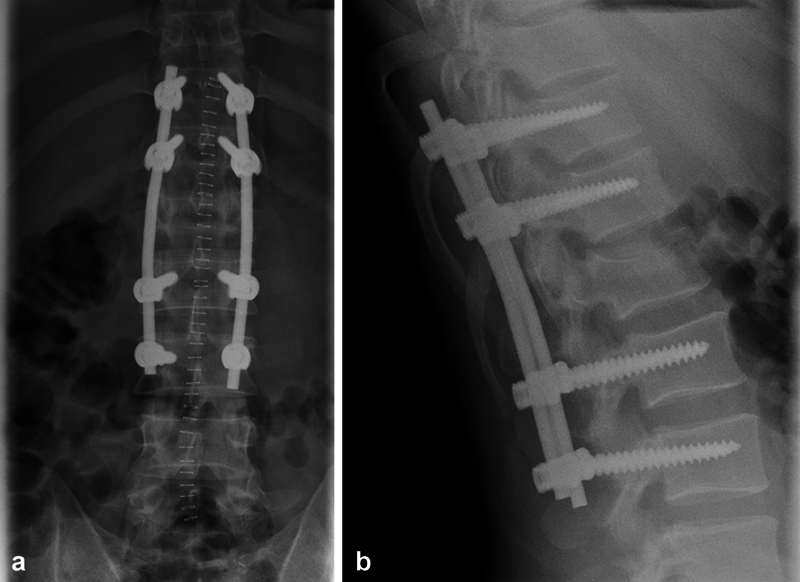
( **a** ) Example of surgical treatment in group I (stand-alone posterior stabilization). ( **b** ) Example of surgical treatment in group I (stand-alone posterior stabilization).

**Fig. 2:**
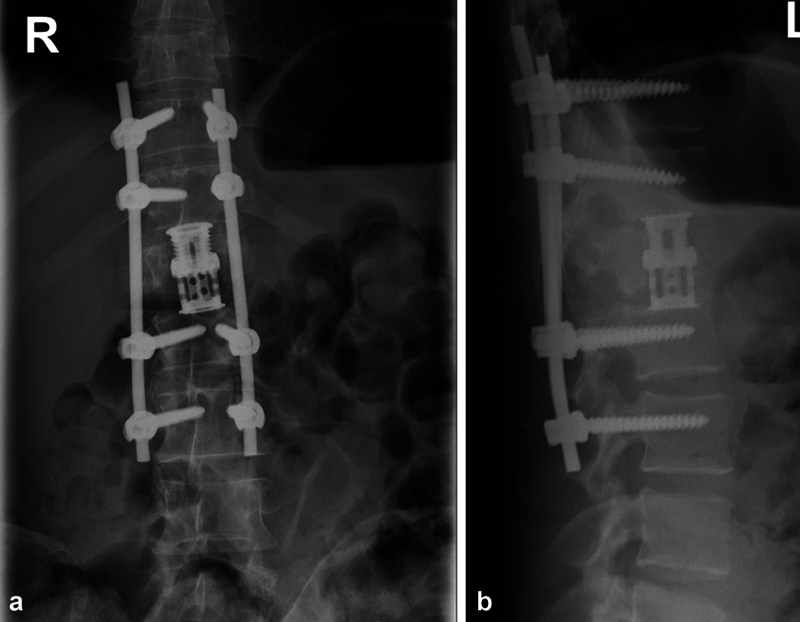
( **a** ) Example of surgical treatment in group II (combined approach). ( **b** ) Example of surgical treatment in group II (combined approach).

The cohort was divided into two groups based on the surgical approach: group I underwent stand-alone posterior dorsal stabilization using transpedicular screws and rods (as exemplified in Fig. 1 ), while group II underwent dorsal stabilization as in group I, followed by a second surgical session within less than 2 weeks, involving anterior replacement of the fractured vertebra through a titanium intervertebral cage (as exemplified in Fig. 2 ).

### Surgical Techniques

After initial clinical and radiologic assessment and cardiopulmonary stabilization of patients in the emergency department, patients were prepared to undertake the surgery. The posterior approach was done in the prone position with a midline incision over the fractured vertebra to undertake decompression, if necessary. Pedicle screw implantation was performed either through open technique by expanding the midline incision to involve two segments above and two segments below the fracture or through percutaneous technique by small paravertebral incisions. No screws were inserted in the fractured segment. The anterior approach was made in a lateral left-sided position through the transthoracic corridor when fractures were in the thoracic or thoracolumbar regions (i.e., from T1 to L1) or through the retroperitoneal corridor when the fractures were in the lumbar region (i.e., from L2 to L4); patients with L5 fractures were operated on through a supine position with an infraumbilical midline incision with the aid of a vascular surgeon. The anterior approach entailed partial resection of the fractured vertebra and implantation of an intervertebral cage filled with autogenous bone.

### Outcomes Measurement

Two spine surgeons independently assessed the radiologic outcomes. Measurements included the SI using the Farcy method 4 and the LVH index estimated through the Keene method. 5 Surgeons independently entered measurements into the dataset based on computed tomography (CT) or X-rays to avoid mutual interference.

**Fig. 3:**
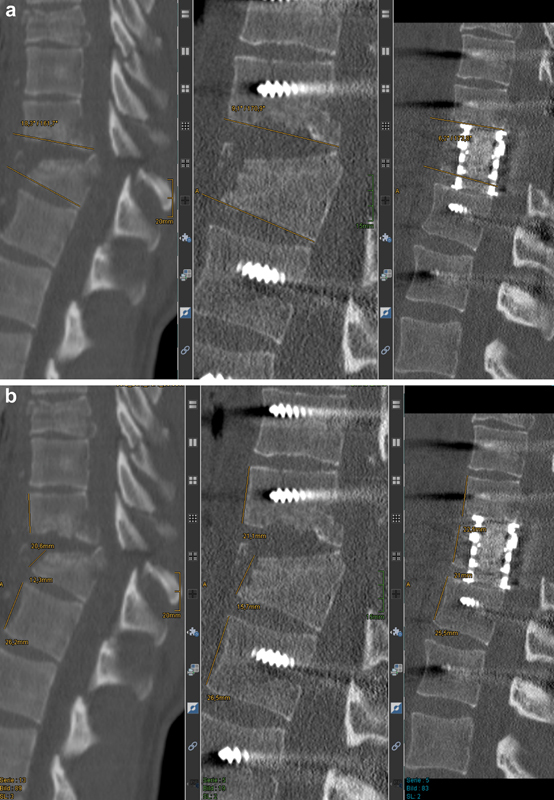
Measurement of the sagittal index (SI) and loss of vertebral height (LVH) in a 28-year-old patient with a T10 fracture after falling from a height (no suicide). ( **a** ) The measurement of SI (left image at presentation = 18.3 + 5= 23.5 degrees; middle image after dorsal stabilization = 9.1 + 5 = 14.1 degrees; and right image after the combined approach = 6.2 + 5= 11.2 degrees). ( **b** ) The measurement of LVH (left image at presentation = 12.3/0.5 (20.6 + 26.2) = 0.526; middle image after dorsal stabilization = 15.7/0.5 (21.1 + 26.5) = 0.660; and right image after the combined approach = 21/0.5 (22.5 + 25.5) = 0.875).

The calculation of the SI involved determining the kyphotic angle at the fractured motion segment level minus the normal contour. Baseline values of 5 degrees in the thoracic region, 0 degrees at the thoracolumbar junction, and –10 degrees in the lumbar region were applied. An SI of zero indicated normal perfect alignment, and any deviation, whether smaller or greater than zero, was recorded as the absolute value. This approach allowed for a standardized scale across all spine regions, indicating malalignment. 4 Fig. 3a illustrates an example of the SI measurement.

LVH was defined as the ratio of the anterior height of the injured vertebra to the mean anterior height of the two adjacent intact vertebrae. An LVH of 1 represented the standard perfect height, and any value smaller than that indicated a loss of height, with zero being the lower limit of LVH. 5 Fig. 3b provides an example of the LVH measurement.

Both the SI and LVH were measured at four time points: baseline (at presentation in groups I and II), after posterior approach (in groups I and II), after the combined approach (in group II) using CT scans in the supine position, and at a follow-up of a minimum of 3 months (in groups I and II) using mostly X-rays in the standing position, if possible, or using CT scans. The number of patients with follow-up X-rays was comparable between both groups (19 in group I vs. 22 in group II).

Postoperative complications were recorded in both groups. Complications were defined as any postoperative condition related to surgical treatment requiring specific treatment or readmission to the hospital.

### Statistical Analysis

Continuous variables were presented as mean ± standard deviation (SD) or median and interquartile range (IQR). The SI and LVH comparisons between groups employed mixed-effects regression modeling, adjusted for baseline values. The mean values of the independent measurements of spine surgeons were used for the SI and LVH. Statistical significance was set at a *p* -value of 0.05. All analyses were performed using StataCorp (2020) Stata Statistical Software: Release 17 (StataCorp LLC, College Station, Texas, United States).

## Results

### Baseline Characteristics

Using the ICD-10 codes S22.0 and S32.0, we identified 135 patients. Among them, 64 were deemed ineligible due to nontraumatic fractures or nonsurgical treatment. Consequently, 71 patients (29 females and 42 males) with a median age of 38 years (IQR: 16–72) were included in the study. Thirty-two patients were assigned to group I (stand-alone posterior stabilization) and 39 patients to group II (combined approach). The median follow-up was 4 months (IQR: 3–17) with a minimum of 3 months. Complete follow-up data were available for 31 patients. Table 1 presents the baseline characteristics of the two groups. Notably, no statistically significant differences in demographic and clinical parameters were observed between the two groups.

**Table 1:** Baseline characteristics in patients with spine injuries

Variable	Group I (stand-alone stabilization), *N* = 32	Group II (combined approach), *N* = 39
Age (mean ± SD), y	41 ± 16	42 ± 19
Sex (female/male)	10/22	19/20
**Cause of injury**		
Suicide jumper Falling Traffic Other trauma	8 12 10 2	9 14 11 5
**Region of injury**		
Thoracic ( *n* ) Thoracolumbar ( *n* ) Lumbar ( *n* )	5 19 8	7 23 9
**AO classification**		
A ( *n* ) B ( *n* ) C ( *n* )	30 2 0	33 4 2
Initial hemoglobin (mean ± SD), g/dL	12.50 ± 2.53	13.12 ± 1.62
Baseline SI (mean ± SD), degrees	11.58 ± 5.85	10.24 ± 6.44
Baseline LVH (mean ± SD), degrees	0.76 ± 0.17	0.71 ± 0.20
Follow-up (median, IQR), mo	4 (3–13)	6 (3–18)

Abbreviations: IQR, interquartile range; LVH, loss of vertebral height; SD, standard deviation; SI, sagittal index.

### Outcomes

**Fig. 4:**
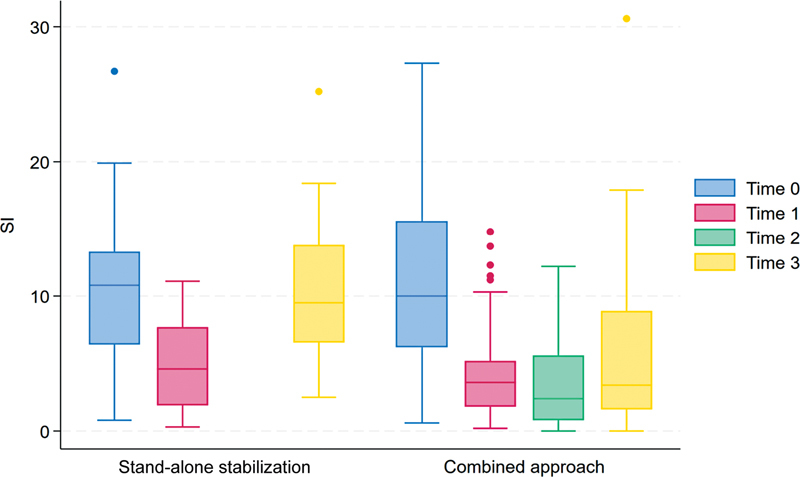
The two groups' sagittal index (SI) at different time points. Time 0: at baseline; time 1: after dorsal approach; time 2: after adding anterior vertebral replacement (in the combined approach group); time 3: at follow-up (a minimum of 3 months).

The baseline SI showed a remarkable improvement (reduction) after dorsal stabilization in both groups (see Fig. 4 ). Group II exhibited a slight additional improvement after the anterior replacement of the fractured vertebra. However, there was a deterioration (increased SI) at the follow-up, which was more pronounced in group I, indicating a regression in sagittal alignment (see Fig. 4 ).

**Fig. 5:**
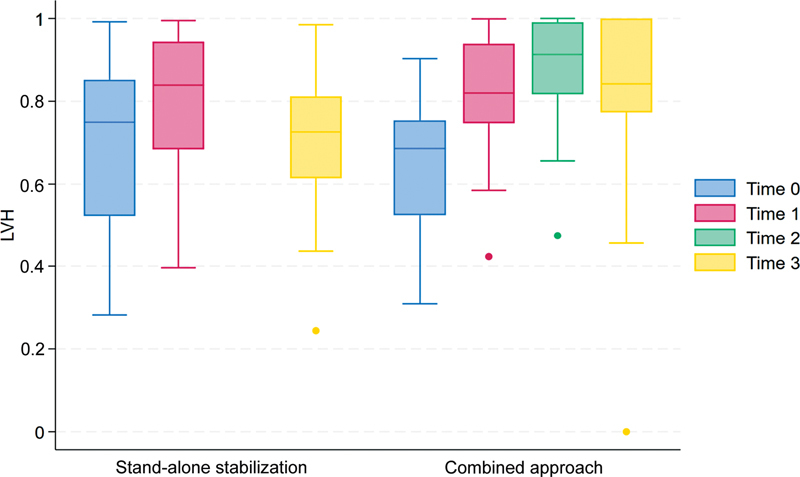
The two groups' loss of vertebral height (LVH) at different time points. Time 0 = at baseline; time 1: after dorsal approach; time 2: after adding anterior vertebral replacement (in the combined approach group); time 3: at follow-up (a minimum of 3 months).

A similar pattern of improvement after each surgical intervention, followed by a subsequent deterioration at follow-up, was observed for the LVH (LVH increased initially after each intervention and then reduced at the follow-up) in both groups (see Fig. 5 ).

A comparison of these outcomes (SI and LVH) through the mixed-effects regression model (see Table 2 ) revealed statistically significant differences between the groups. Group II showed a slight improvement in both indices at the follow-up compared with group I, indicating a better sagittal alignment and slightly more effective and significantly more lasting restoration of vertebral height through the combined approach.

**Table 2:** Radiologic outcomes (SI and LVH) at follow-up per mixed regression analysis with adjustment to baseline values of SI and LVH between the two approaches

Outcome	Adjusted mean difference (group II – group I) a	95% CI	*p* -value
SI	–4.24 b	–7.13 to –1.36	0.004
LVH	0.11 b	0.02–0.20	0.02

Abbreviations: CI, confidence interval; LVH, loss of vertebral height; SI, sagittal index.

a Mean difference (SI in group II minus SI in group I) at follow-up calculated through a mixed regression model taking all outcome measurements at different time points in the model with adjustment to the baseline values.

b SI difference is negative, indicating that group II has less malalignment since the best SI should be zero. On the other hand, LVH is positive, indicating that group II has a better vertebral height since the best LVH is 1 (i.e., no loss of vertebral height).

Regarding complications, there were four cases in group I and five in group II. A detailed breakdown of all complications in both groups is provided in Table 3 . The complications in group II were mainly pulmonary related because of the transthoracic corridor when undertaking the anterior approach in patients with fractures in the thoracic or thoracolumbar junction regions. In contrast, the main complications in group I were related specifically to the insufficiency of fixation materials in patients with fractures in the thoracolumbar or lumbar regions.

**Table 3:** Postoperative complications in two groups

Group I (stand-alone posterior stabilization)	Vertebra	Group II (combined approach)	Vertebra
1. Chronic back pain 2. Reoperation because of pedicle screw insufficiency 3. Reoperation because of the shortage of the fixation system 4. Reoperation because of fixation system insufficiency	L1 T11 L1 L2	1. Pneumothorax 2. Pneumonia. 3. Wound infection 4. Reoperation because of insufficiency of the fixation system 5. Lung embolism	T9 L1 L2 L4 T12

## Discussion

In 1959, Boucher reported the pioneering use of pedicle screws for treating spine fractures. This technique marked the inception of a treatment approach that has since evolved in technique and material. 7 8 The replacement of the vertebral body with foreign material to treat spine fractures was first described in 1967 by Scoville and others, and since then, it has undergone further development. 9 The optimal approach for managing unstable spine fractures remains a point of contention for spine surgeons. 8 10 Our study comparing the stand-alone posterior and combined anteroposterior approaches showed significant differences in radiologic outcomes. Specifically, this concerned the SI and LVH. Intriguingly, postoperative complications did not exhibit a statistically significant difference during follow-up. Less efficient restoration of alignment and vertebral height may cause apparent clinical problems later.

Singh et al previously suggested that adding an anterior reconstruction to the stand-alone posterior approach could enhance the correction of sagittal alignment and LVH. 11 However, their study focused on a small subgroup of patients ( *n* = 4), limiting generalizability. Another case series, albeit without a control group, echoed positive results for posterior/anterior combined surgery. 10 Our analysis, incorporating a control group and a relatively large cohort ( *n* = 39 in the combined approach and *n* = 32 in the dorsal approach), substantiates these prior findings statistically and methodologically.

On the contrary, a randomized controlled trial (RCT) that compared the combined approach with the stand-alone posterior stabilization in 40 patients observed no difference in radiologic deformity correction and clinical outcomes. 12 Although this study is an RCT, there are two points of criticism to be mentioned here: First, the authors included only patients with L2, L3, or L4 fractures, which limits the generalizability of their results on other spinal regions. Second, statistically, they compared the preoperative and postoperative radiologic outcomes in the same group, not between the groups. At the final observation in this RCT, the sagittal deformity correction was better in the combined group, 12 which matched our findings at the final follow-up.

In a recent systematic review comparing the two groups, authors found no statistically significant difference in the radiologic outcomes; however, they included only patients with thoracolumbar junction fractures and measured only the Cobb angle to assess the deformity. 13 To the best of our knowledge, this is the first study that compared the SI and LVH in traumatic thoracic or lumbar fractures between these two groups of patients.

Our results, aligning with biomechanical principles, demonstrate that sagittal alignment and vertebral height improvement were comparable in both groups in the early postoperative period when the patients were less mobile. However, after discharge and a minimum of 3-month follow-up, a decline in radiologic outcome was noted, particularly in the stand-alone posterior stabilization group. This suggests that increased loading forces postoperatively compromised the stability of the weakest fractured region, with the combined approach exhibiting superior outcomes through an increase in the load-carrying capacity of the spine. Consistent with the biomechanical properties of the thoracolumbar spine, our recommendation emphasizes reconstructing all three columns for severe spine fractures in the thoracic or lumbar regions. 14

Acknowledging limitations, our study faced challenges with follow-up measurements for about half the patients. However, using a mixed-effects model helped address this limitation through multiple imputations. 15 Another limitation concerns the follow-up period, which was short in most patients, so the effect of the constructs in both approaches under long-term loading could have been different. The construct in the combined approach is more rigid, which can lead to more adjacent segment problems with resulting deformity. On the other hand, less restored SI and LVH in stand-alone posterior stabilization can also increase the adjacent segment problem with resulting kyphosis. In light of this limitation, another follow-up study with longer follow-up is warranted. Moreover, the extended follow-up period, ranging from 3 months to over 3 years, contributed to outcome variability.

While we found statistically significant differences, we acknowledge that these primarily pertained to radiologic outcomes, serving as surrogates for clinical significance. Nevertheless, examination of postoperative complications revealed that stand-alone posterior stabilization was more susceptible to implant failure, which might carry a risk of clinical deterioration at a later time point. Expanding our study by involving a larger sample size and longer follow-up with registration of additional clinical outcomes will address these limitations in future research.

## Conclusions

In addressing severe spine fractures within the thoracic and lumbar regions, optimal surgical treatment should prioritize the restoration of alignment and height. Our findings support the consideration of an anterior replacement of the vertebra in conjunction with posterior stabilization using pedicle screws, commonly known as a combined anteroposterior approach. Compared with stand-alone posterior stabilization, this combined approach demonstrates superiority in maintaining a more favorable sagittal alignment and vertebral height at follow-up. These conclusions emphasize the potential benefits of a comprehensive three-column reconstruction strategy for better outcomes in managing severe thoracic and lumbar spine fractures.
